# Proteasome 26S subunit, non-ATPases 1 (PSMD1) and 3 (PSMD3), play an oncogenic role in chronic myeloid leukemia by stabilizing nuclear factor-kappa B

**DOI:** 10.1038/s41388-021-01732-6

**Published:** 2021-03-12

**Authors:** Alfonso E. Bencomo-Alvarez, Andres J. Rubio, Idaly M. Olivas, Mayra A. Gonzalez, Rebecca Ellwood, Carme Ripoll Fiol, Christopher A. Eide, Joshua J. Lara, Christian Barreto-Vargas, Luis F. Jave-Suarez, Georgios Nteliopoulos, Alistair G. Reid, Dragana Milojkovic, Brian J. Druker, Jane Apperley, Jamshid S. Khorashad, Anna M. Eiring

**Affiliations:** 1 Center of Emphasis in Cancer, Department of Molecular and Translational Medicine, Paul L. Foster School of Medicine, Texas Tech University Health Sciences Center El Paso, El Paso, TX USA; 2 Graduate School of Biomedical Sciences, Texas Tech University Health Sciences Center El Paso, El Paso, TX USA; 3 Centre for Haematology, Department of Medicine, Imperial College London, London, UK; 4 Knight Cancer Institute, Division of Hematology/Medical Oncology, Oregon Health & Science University, Portland, OR USA; 5 Universidad de Guadalajara, Guadalajara, Jalisco México; 6 Instituto Mexicano del Seguro Social, Centro de Investigaciόn Biomédica de Occidente, Guadalajara, Jalisco México; 7 Department of Surgery and Cancer, Imperial College London, London, UK; 8 Molecular Pathology Unit, Liverpool Clinical Laboratories, Royal Liverpool University Hospital, Liverpool, UK

**Keywords:** Chronic myeloid leukaemia, Oncogenes

## Abstract

Tyrosine kinase inhibitors (TKIs) targeting BCR-ABL1 have revolutionized therapy for chronic myeloid leukemia (CML), paving the way for clinical development in other diseases. Despite success, targeting leukemic stem cells and overcoming drug resistance remain challenges for curative cancer therapy. To identify drivers of kinase-independent TKI resistance in CML, we performed genome-wide expression analyses on TKI-resistant versus sensitive CML cell lines, revealing a nuclear factor-kappa B (NF-κB) expression signature. Nucleocytoplasmic fractionation and luciferase reporter assays confirmed increased NF-κB activity in the nucleus of TKI-resistant versus sensitive CML cell lines and CD34^+^ patient samples. Two genes that were upregulated in TKI-resistant CML cells were proteasome 26S subunit, non-ATPases 1 (*PSMD1*) and 3 (*PSMD3*), both members of the 19S regulatory complex in the 26S proteasome. *PSMD1* and *PSMD3* were also identified as survival-critical genes in a published small hairpin RNA library screen of TKI resistance. We observed markedly higher levels of *PSMD1* and *PSMD3* mRNA in CML patients who had progressed to the blast phase compared with the chronic phase of the disease. Knockdown of PSMD1 or PSMD3 protein correlated with reduced survival and increased apoptosis in CML cells, but not in normal cord blood CD34^+^ progenitors. Luciferase reporter assays and immunoblot analyses demonstrated that PSMD1 and PSMD3 promote NF-κB protein expression in CML, and that signal transducer and activator of transcription 3 (STAT3) further activates NF-κB in scenarios of TKI resistance. Our data identify NF-κB as a transcriptional driver in TKI resistance, and implicate PSMD1 and PSMD3 as plausible therapeutic targets worthy of future investigation in CML and possibly other malignancies.

## Introduction

Chronic myeloid leukemia (CML) is a malignancy of the pluripotent hematopoietic stem cell, in which a reciprocal translocation between chromosomes 9 and 22 produces BCR-ABL1, the oncogenic tyrosine kinase that drives disease [1]. In newly diagnosed CML patients, tyrosine kinase inhibitors (TKIs) targeting BCR-ABL1 are remarkably effective at eliminating most *BCR-ABL1*-positive cells, especially in the chronic phase (CP-CML) [2, 3]. However, TKIs do not eliminate CML leukemic stem cells (LSCs) [4–7], and while some studies have reported treatment-free remission following deep molecular response [8–10], life-long therapy is required to maintain remission in most patients [11, 12]. Long-term TKI treatment is associated with a high economic burden and a reduced quality of life, including adverse cardiovascular events [13–16] and skeletal muscle toxicity [17]. Additionally, patients who progress to the blast phase of CML (BP-CML) still have a poor prognosis [2, 3]. Understanding the mechanisms driving TKI resistance will inform treatment strategies aimed at curing the disease.

TKI resistance is often linked to point mutations in the BCR-ABL1 kinase domain that impair drug binding [18]. However, many cases of clinical resistance occur in the absence of *BCR-ABL1* mutations [3]. BCR-ABL1-independent resistance is a feature of the CML LSC population, providing a reservoir of cells for disease recurrence via mechanisms that are not well understood [5, 6, 19]. Our previous work demonstrated that CML stem and progenitor cells that are TKI-resistant, but lack explanatory BCR-ABL1 kinase domain mutations, are dependent on the activation of alternative signaling pathways, especially signal transducer and activator of transcription 3 (STAT3) [19–21]. To better understand the transcriptional drivers of TKI resistance, we performed genome-wide expression analyses on TKI-resistant CML cells versus parental controls using RNA sequencing (RNA-seq). Analysis of this data set implicated nuclear factor-kappa B (NF-κB) as the transcriptional driver of TKI resistance. NF-κB is known to regulate the expression of diverse gene targets in many different cancers, including CML [22–24].

The ubiquitin-proteasome system (UPS) plays an important role in activating and terminating NF-κB signaling in many different scenarios [25, 26]. Interestingly, two members of the UPS, proteasome 26S subunit, non-ATPases 1 (PSMD1) and 3 (PSMD3), were upregulated in TKI-resistant CML cell lines and patient samples [27, 28]. They were also identified as survival-critical genes in a previously published small hairpin RNA (shRNA) library screen for TKI resistance [29]. PSMD1 and PSMD3 are members of the 19S regulatory complex in the 26S proteasome, regulating substrate recognition and binding [30, 31]. In breast cancer, PSMD1 promoted cancer cell growth by inducing p53 protein degradation [30]; PSMD3, in contrast, enhanced cancer cell growth by stabilizing human epidermal growth factor receptor 2 (HER2) from degradation [31]. In acute myeloid leukemia (AML), patients with higher levels of *PSMD3* mRNA were shown to have a worse overall survival than patients with lower levels of expression [32]. In the present study, we hypothesized that PSMD1 and PSMD3 mediate NF-κB activation in CML and TKI resistance. Here, we report that PSMD1 and PSMD3 promote NF-κB protein expression and transcriptional activity in CML, and that STAT3 perpetuates this signal in scenarios of TKI resistance. Our data implicate PSMD1 and PSMD3 as potential targets for combination therapies in myeloid malignancies and possibly other cancers.

## Results

### The transcriptional signature of TKI resistance is reminiscent of tumor necrosis factor alpha (TNFα) signaling via NF-κB

**Fig. 1:**
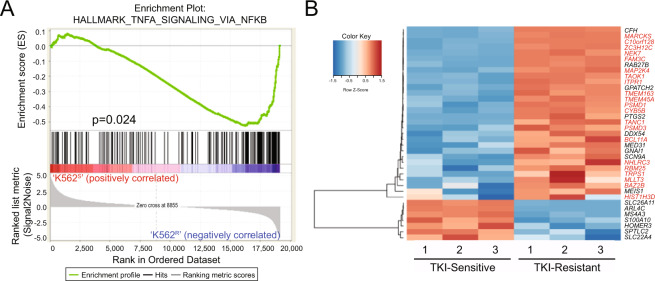
Imatinib resistance associates with an NF-κB expression signature. **A** RNA from K562^S^ and K562^R^ cells were analyzed by RNA-seq to assess for differences in gene expression (*n* = 3 samples/group). GSEA [77, 78] implicated the TNFα signaling pathway via NF-κB (FWER: *p* = 0.024). **B** The heat map shows genes that were commonly dysregulated in both TKI-resistant cell lines and patient samples [27], identifying 30 upregulated genes and 7 downregulated genes. Of the 30 upregulated genes, 21 are predicted to harbor NF-κB binding sites in and around the promoter region (indicated in red).

To investigate potential drivers of BCR-ABL1 kinase-independent TKI resistance, we performed RNA-seq on extracts from TKI-resistant K562^R^ cells versus parental, TKI-sensitive K562^S^ controls. K562^R^ cells are an in vitro model of BCR-ABL1-independent TKI resistance, which lack explanatory *BCR-ABL1* kinase domain mutations and survive despite TKI-mediated BCR-ABL1 inhibition [19, 21]. An MA plot was produced from DESeq2 to visualize the data (Supplementary Fig. S1A), and Euclidean distance calculations demonstrated clear separation between the two cell lines (Supplementary Fig. S1B). Gene set enrichment analysis (GSEA) [22–24] on the differentially expressed genes implicated TNFα signaling via NF-κB as the top pathway (*p* = 0.024) (Fig. 1A). Protein functions represented by the top differentially expressed genes (Supplementary Fig. S1C) included apoptosis, angiogenesis, proliferation, ubiquitylation, and transcription (Supplementary Table S4).

We correlated the fold change of gene expression in our RNA-seq data with that of a previously published microarray study comparing CD34^+^ cells from CP-CML patients who either responded or did not respond to imatinib [27]. Integration of genome-wide expression analyses with microarray data from this study identified 37 genes commonly dysregulated in TKI-resistant CML cell lines and patient samples (Fig. 1B and Supplementary Table S5). Of the 30 genes that are upregulated in TKI resistance, 21 are predicted to be NF-κB transcriptional targets based on data available from the University of California Santa Cruz Genome Browser (lymphoblastoid cell lines treated with TNFα, data deposited by Michael Snyder’s lab at Stanford University) (Fig. 1B) [33, 34]. Therefore, NF-κB may be driving the gene expression signature of TKI resistance.

### Nuclear NF-κB transcriptional activity is increased in TKI-resistant CML cell lines and primary CML CD34^+^ cells

**Fig. 2:**
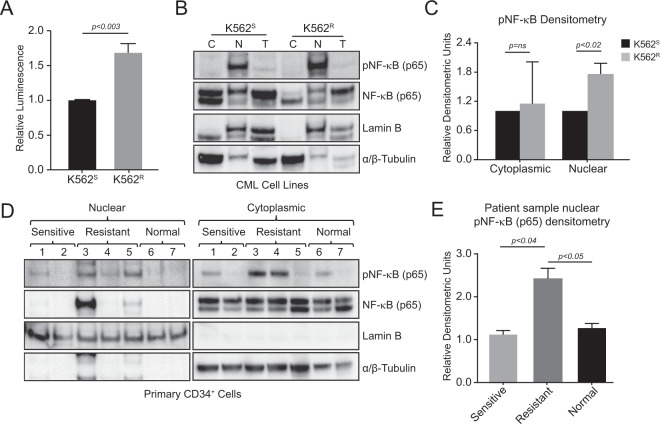
Transcriptionally active NF-κB is increased in the nucleus of TKI-resistant CML cells. **A** Bar graphs represent relative luciferase reporter activity in K562^S^ versus K562^R^ cells (*n* = 5 samples/group) stably transduced with an NF-κB reporter versus a negative control. **B** Immunoblot shows NF-κB and pNF-κB in the nucleus and cytoplasm of K562^S^ versus K562^R^ cells. **C** Bar graph represents densitometric calculations for separate replicates (*n* = 3) of the nucleocytoplasmic fractionation experiments presented in panel (**B**). Densitometric analysis was completed by normalizing against lamin B and tubulin in the nuclear and cytoplasmic fractions, respectively. **D** Immunoblot shows NF-κB and pNF-κB in the nucleus and cytoplasm of CD34^+^ cells from newly diagnosed, TKI-sensitive CP-CML patients (*n* = 2) versus TKI-resistant patients (*n* = 3) and normal donors (*n* = 2). Lamin B and tubulin were used to assess the quality of separation of the nuclear and cytoplasmic fractions, respectively. **E** Bar graph represents densitometric calculations for pNF-κB in panel (**D**). Error bars represent standard error of mean (SEM). C cytoplasmic fraction, N nuclear fraction, T total fraction.

It is well documented that NF-κB contributes to drug resistance in many different cancers [35–38]. In CML, NF-κB is activated by proteins like COBL11 [39] and TPL2 [40], and NF-κB inhibition was shown to sensitize TKI-resistant CML cells to imatinib [41–44]. However, the functional role of NF-κB transcriptional activity and the mechanism of activation in TKI resistance has not been explored. Confirming our RNA-seq data linking NF-κB with TKI resistance, TKI-resistant K562^R^ cells demonstrated a 70% increase of NF-κB-mediated luciferase reporter activity compared to controls (Fig. 2A). Accordingly, nucleocytoplasmic fractionation followed by immunoblot analyses revealed a 75% increase of pNF-κB in the nucleus of K562^R^ cells compared with controls (Fig. 2B, C). Similar results were seen in primary, TKI-resistant CML CD34^+^ cells. We observed a twofold increase of pNF-κB levels in the nucleus of cells from two of three TKI-resistant CML patients compared with newly diagnosed CP-CML patients and normal donors (Fig. 2D, E). These data implicate a role for enhanced NF-κB transcriptional activity in driving the gene expression signature of TKI resistance in CML cell lines and patients who fail imatinib therapy.

### PSMD1 and PSMD3 expression is upregulated during CML disease progression

It is well established that the UPS influences NF-κB activation and transcriptional activity [25, 26]. We previously performed a functional genomics study using a pooled shRNA library on K562^R^ versus K562^S^ cells. This approach identified five members of the UPS as genes critical to survival in TKI resistance, including *NEDD8*, *PSMA1*, *PSMD1*, *PSMD3*, and *UBE1* [29]. Therefore, we searched for genes related to the UPS in our list of commonly dysregulated TKI resistance genes (Fig. 1 and Supplementary Table S5), identifying *PSMD1* and *PSMD3*. PSMD1 and PSMD3 are non-ATPase subunits in the 19S regulatory complex of the UPS, which regulate proteasome substrate recognition and binding [30, 31]. To date, the role of PSMD1 and PSMD3 in CML and NF-κB activation remains unknown.

**Fig. 3:**
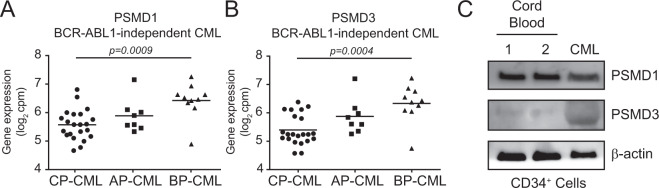
PSMD1 and PSMD3 are upregulated during CML disease progression. RNA-seq data demonstrated increased *PSMD1* (**A**) and *PSMD3* (**B**) mRNA levels in mononuclear cells from BP-CML (*n* = 10) compared to CP-CML (*n* = 13) and AP-CML (*n* = 8) patients demonstrating BCR-ABL1-independent TKI resistance. **C** Immunoblot shows PSMD1 and PSMD3 protein levels in CD34^+^ cells from cord blood (lanes 1–2) versus a CP-CML patient (lane 3). β-actin was assessed as a loading control.

RNA-seq data using specimens from CML patients with primary BCR-ABL1-independent resistance revealed that both *PSMD1* and *PSMD3* are upregulated in BP-CML patient samples compared with patients in the chronic or accelerated phases of the disease (Fig. 3A, B). These data are consistent with previously published microarray data on primary CML CD34^+^ cells by Radich et al. [28], indicating that *PSMD1* and *PSMD3* are upregulated during CML disease progression. Immunoblot analysis on primary CD34^+^ cells from normal cord blood versus that of a CML patient demonstrated that PSMD3 but not PSMD1 protein is upregulated in CML samples compared to normal controls (Fig. 3C). Thus, we hypothesized that PSMD3, and possibly PSMD1, may play a role in NF-κB activation during CML disease progression and imatinib resistance.

### Knockdown of PSMD1 or PSMD3 reduced survival and increased apoptosis in CML but not normal hematopoietic progenitor cells

**Fig. 4:**
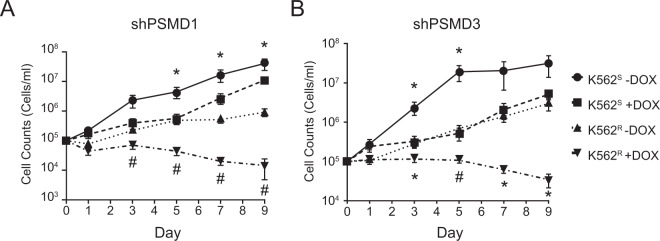
Knockdown of the proteasome components, PSMD1 or PSMD3, resulted in a greater reduction of growth in TKI-resistant compared with TKI-sensitive K562 cells. Line graphs show cell growth in K562^S^ (*n* = 3) and K562^R^ cells (*n* = 4) expressing shRNA targeting PSMD1 (shPSMD1, (**A**)) or PSMD3 (shPSMD3, (**B**)) in the presence and absence of doxycycline (100 ng/ml) to induce the knockdown. Counts were recorded and graphed on a log scale. Error bars represent SEM. *p < 0.05; ^#^*p* < 0.01.

The proteasome inhibitor, bortezomib, was previously shown to induce apoptosis of primitive, TKI-resistant CML LSCs [45]. However, there was little efficacy and considerable toxicity observed in CML patients receiving bortezomib therapy [46, 47]. We reasoned that inhibition of PSMD1 or PSMD3 might represent a novel method for proteasome inhibition with less toxicity. To confirm the findings of our shRNA library screen [29], K562^S^ and K562^R^ cells were stably transduced with shRNA targeting *PSMD1* (shPSMD1) or *PSMD3* (shPSMD3). Using the same constructs that were hits in the shRNA library screen [29], we confirmed a 50–80% knockdown at the mRNA and protein level upon doxycycline-induced knockdown (Supplementary Fig. S2A, B). Consistent with the library screen [29], shPSMD1 and shPSMD3 impaired the growth of K562^R^ cells by 9.5-fold and 4.6-fold more than in K562^S^ cells, respectively, over the 9-day period (Fig. 4A, B).

**Fig. 5:**
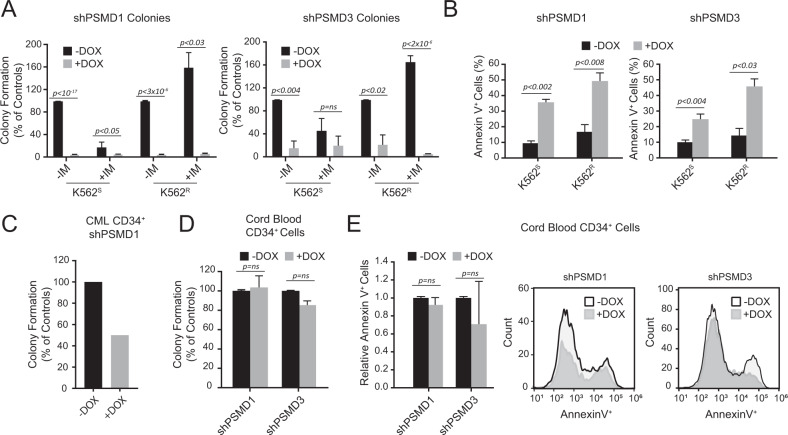
shRNA-mediated knockdown of PSMD1 or PSMD3 impairs survival and induces apoptosis in CML but not normal progenitor cells. Bar graphs represent the effects of shPSMD1 or shPSMD3 on colony forming ability (**A**) or AnnexinV positivity (**B**) in K562^S^ and K562^R^ cells with and without imatinib (1 μM) or doxycycline (100 ng/ml) as indicated (at least three samples were analyzed for all groups tested). **C** Bar graph represents the effects of shPSMD1 on colony formation of CD34^+^ cells from a CP-CML patient cultured ± doxycycline (100 ng/ml) to induce the knockdown. SARS-CoV-2 2019 (COVID-19) prevented completion of these experiments in additional patient samples. **D** Bar graph represents colony forming ability (*n* = 5 samples/group) of cord blood CD34^+^ cells expressing shPSMD1 or shPSMD3 in the presence and absence of doxycycline. **E** Bar graph and histograms demonstrate AnnexinV positivity in cord blood CD34^+^ cells expressing shPSMD1 or shPSMD3 in the presence and absence of doxycycline (*n* = 2).

To further understand the role of PSMD1 and PSMD3 in CML and TKI response, we assessed the effects of shPSMD1 and shPSMD3 on colony forming ability and apoptosis in K562^S^ and K562^R^ cells compared with a non-targeting control vector (shNT). As expected, shNT had no effect on *PSMD1* or *PSMD3* mRNA or protein levels and did not alter colony forming ability in parental K562 cells (Supplementary Fig. S3A–C). PSMD1 and PSMD3 knockdown nearly ablated colony forming ability in both cell lines in the presence and absence of imatinib, which correlated with the induction of apoptosis (Fig. 5A, B). Knockdown of PSMD1 also reduced colony formation in CD34^+^ cells from a CP-CML patient by 50% (Fig. 5C). Similar experiments were performed in CD34^+^ cells harvested from normal cord blood. In contrast to our results in CML cells, knockdown of PSMD1 and PSMD3 had no effect on colony formation or apoptosis of cord blood CD34^+^ cells (Fig. 5D, E), suggesting a therapeutic window that could potentially be exploited.

**Fig. 6:**
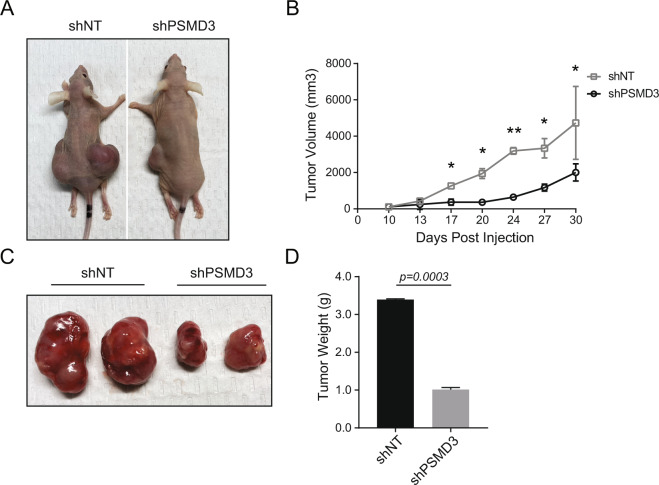
Knockdown of PSMD3 reduces K562 tumor formation in a subcutaneous model of CML. **A** Representative nude mice displaying tumors resulting from subcutaneous injection of K562 cells expressing either the shNT control (left) or shPSMD3 (right). **B** Line graph shows the rate of tumor growth of K562 cells transduced with shNT versus shPSMD3 implanted into the rear flanks of nude mice (*n* = 2/group). Image and bar graph shows tumor sizes (**C**) and weights (**D**). Error bars represent the mean (*p < 0.05; **p < 0.01).

Since PSMD3 and not PSMD1 was upregulated in CML CD34^+^ cells (Fig. 3C), we assessed whether PSMD3 knockdown altered K562 proliferation in vivo. K562 cells transduced with shPSMD3 or the shNT control were embedded in Matrigel and implanted into the rear flanks of nude mice. Ten days following injection, mice were placed on doxycycline chow to induce vector expression, and tumor size was evaluated over time. Knockdown of PSMD3 resulted in a significant reduction of subcutaneous tumor size by over threefold compared with shNT tumors (Fig. 6A–D). Altogether, our data implicate a role for the proteasome component, PSMD3, and possibly PSMD1, in disease progression and survival in CML.

### Knockdown of PSMD1 or PSMD3 reduced NF-κB protein expression and transcriptional activity in CML cells that are both sensitive and resistant to imatinib

**Fig. 7:**
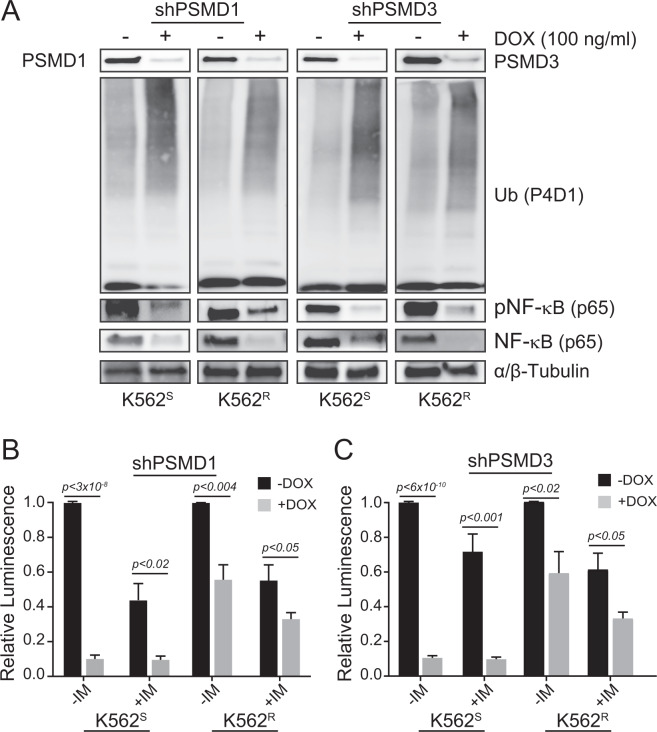
PSMD1 and PSMD3 regulate NF-κB expression and transcriptional activity in TKI-sensitive and TKI-resistant CML cells. **A** Immunoblot shows the effects of shPSMD1 (left) and shPSMD3 (right) on levels of ubiquitylated proteins, total NF-κB p65, and phospho-NF-κB p65 in both K562^S^ and K562^R^ cells upon knockdown of PSMD1 and PSMD3 protein (*n* = 3 samples/group). Bar graphs represent NF-κB-driven luciferase reporter activity in K562^S^ and K562^R^ cells lentivirally transduced with shPSMD1 (**B**) (*n* = 4) or shPSMD3 (**C**) (*n* = 3) ± doxycycline (100 ng/ml, 72 h) to induce the knockdown. Error bars represent SEM.

To determine whether PSMD1 or PSMD3 regulates NF-κB activation in CML and TKI resistance, we used shRNAs to knockdown their expression in TKI-sensitive versus TKI-resistant CML cells. Knockdown of PSMD1 or PSMD3 protein resulted in global accumulation of ubiquitylated proteins in both cell lines, suggesting impaired protein deubiquitylation (Fig. 7A). This correlated with a reduction of both phospho-NF-κB and total NF-κB protein expression (Fig. 7A). Densitometry revealed no difference in the relative reduction of NF-κB protein in TKI-resistant versus TKI-sensitive K562 cells in response to knockdown of either protein (Supplementary Fig. S4A, B). Consequently, shPSMD1 and shPSMD3 reduced NF-κB luciferase reporter activity by 90% in K562^S^ cells, but only 50% in K562^R^ cells, in the presence and absence of imatinib (Fig. 7B, C). These data indicate that PSMD1 and PSMD3 promote UPS-dependent NF-κB activation in CML, and suggest that other factors are responsible for further activating NF-κB during TKI resistance.

Next we assessed the sensitivity of K562^S^ versus K562^R^ cells to graded concentrations of the proteasome inhibitor, bortezomib, and found that TKI-resistant cells demonstrated cross-resistance to proteasome inhibition (Supplementary Fig. S5A). However, bortezomib reduced relative NF-κB luciferase reporter activity to a similar degree in both cell lines (Supplementary Fig. S5B). Altogether, these data suggest a potential role for alternative signaling pathways in activating NF-κB during TKI resistance.

### STAT3 and not TNFα is responsible for activating NF-κB in TKI-resistant CML

**Fig. 8:**
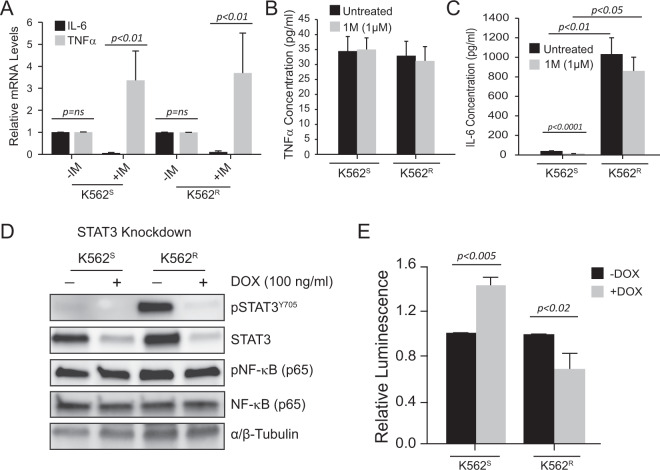
A role for IL-6 and STAT3 in activation of NF-κB transcriptional activity in TKI resistance. **A** Bar graph shows relative *IL6* and *TNFa* mRNA levels in TKI-sensitive K562^S^ cells versus TKI-resistant K562^R^ cells in the presence and absence of imatinib (1 μM, 24 h). Bar graphs represent the concentration of TNFα (**B**) or IL-6 (**C**) produced by K562^S^ versus K562^R^ cells in the presence and absence of imatinib (1 μM, 24 h). **D** Immunoblot shows total STAT3, phospho-STAT3^Y705^, total NF-κB, and phospho-NF-κB in K562^S^ and K562^R^ cells upon induction of shRNA targeting STAT3 (shSTAT3) with doxycycline (100 ng/ml, 72 h). **E** Bar graph represents NF-κB luciferase reporter activity in K562^S^ versus K562^R^ cells upon induction of shSTAT3 with doxycycline (100 ng/ml, 72 h). Error bars represent SEM.

Knockdown of PSMD1 or PSMD3 reduced NF-κB reporter activity by ~90% and ~50% in K562^S^ and K562^R^ cells, respectively (Fig. 7B, C). Therefore, we hypothesized that other factors are responsible for further activation of NF-κB during TKI resistance. Constitutive NF-κB activation upregulates the expression of major inflammatory mediators, including TNFα, interleukin-6 (IL-6), IL-1, and IL-8, to promote cell proliferation and inhibit apoptosis [48, 49]. TNFα and IL-6 have been shown to support stem and progenitor cell survival and proliferation in CML [50–53]. Additionally, several groups have reported a role for cytokines in the CML bone marrow microenvironment [53–57], and blockade of extrinsic survival signals was shown to restore sensitivity of CML cells to TKIs [58, 59]. Since our RNA-seq data implicated TNFα signaling via NF-κB, we hypothesized that autocrine TNFα production was responsible for further NF-κB activation in TKI resistance. TNFα was previously reported to activate NF-κB in AML [60]. We first measured *TNFA* and *IL6* mRNA by reverse transcription quantitative polymerase chain reaction (RT-qPCR) in both K562^S^ and K562^R^ cells either untreated or treated with imatinib. Imatinib treatment markedly increased *TNFA* mRNA expression in both K562^S^ and K562^R^ cells, with no observable differences between the two groups (Fig. 8A). In contrast, *IL6* mRNA expression was completely abolished by imatinib treatment, again with no observable differences between the cell lines (Fig. 8A). Next we performed Enzyme-linked immunosorbent assays (ELISAs) to assess for autonomous TNFα or IL-6 secretion at the protein level. In contrast with our RNA-seq data, TNFα production was low in both K562^S^ and K562^R^ cells in the presence or absence of imatinib, and there was no difference between the two groups (Fig. 8B). IL-6, on the other hand, was significantly reduced by imatinib treatment in K562^S^ cells, and markedly increased in K562^R^ cells under all treatment conditions (Fig. 8C).

Our previous work demonstrated that CML stem and progenitor cells that are TKI-resistant, but lack BCR-ABL1 kinase domain mutations, are dependent on STAT3 and susceptible to STAT3 inhibition [19, 20]. The NF-κB and STAT3 signaling pathways have been shown to functionally interact with each other in many different scenarios [61–66]. To assess whether STAT3 plays a role in NF-κB activation during TKI resistance, we obtained a lentiviral shRNA vector to knockdown STAT3 expression (shSTAT3), and analyzed those cells for changes in NF-κB luciferase reporter activity. We have already reported that shSTAT3 has no effect on survival of TKI-sensitive CML cells, but markedly impairs survival of TKI-resistant cells, especially in the presence of imatinib [19]. shSTAT3 efficiently knocked down STAT3 protein in both K562^S^ and K562^R^ cells (Fig. 8D). In K562^S^ cells, shSTAT3 resulted in a slight upregulation of NF-κB luciferase reporter activity (Fig. 8E). In TKI-resistant cells, however, shSTAT3 significantly reduced NF-κB luciferase reporter activity (Fig. 8E). Altogether, our data suggest that PSMD1 and PSMD3 stabilize NF-κB from degradation in CML, and that STAT3 perpetuates this signal in scenarios of TKI resistance.

## Discussion

Therapies involving TKIs in combination with drugs that eliminate TKI-resistant leukemia stem and progenitor cells would be a major innovation in cancer therapy, leading to a definitive cure for CML patients. Our previous work demonstrated that combined inhibition of STAT3 and BCR-ABL1 induced synthetic lethality in therapy-resistant CML cell lines and patient samples [19, 20]. However, SH2 domain-containing proteins like STAT3 are challenging molecular targets, because inhibition results in off-target effects [67]. To identify drivers of TKI resistance, we performed RNA-seq on TKI-resistant versus parental K562 cells. Surprisingly, GSEA did not reveal a STAT3 transcriptional signature, but was rather reminiscent of TNFα signaling via NF-κB (Fig. 1). In the present study, we elucidated the mechanism by which NF-κB is activated in CML and TKI resistance, implicating two important and novel findings: (1) the non-ATPase proteasome subunits, PSMD1 and PSMD3, are upregulated during CML disease progression (Fig. 3) and promote NF-κB protein expression in CML (Fig. 7), and (2) STAT3 further activates NF-κB in scenarios of TKI resistance (Fig. 8). This is a relevant finding, because it implicates noncanonical functions for STAT3 during drug resistance of CML.

Interestingly, our data suggest that knockdown of PSMD1 or PSMD3 has differential effects on cell growth versus survival signals in CML. In cell growth assays, knockdown of either protein resulted in a greater reduction of growth in TKI-resistant compared with TKI-sensitive cells (Fig. 4). This is consistent with our previous findings in a shRNA library screen [29]. In contrast, PSMD1 or PSMD3 knockdown in either cell line had similar effects on reducing survival in colony formation assays (Fig. 5). This suggests that these proteins differentially control cell growth but not survival signals in TKI resistance. While *PSMD1* and *PSMD3* were identified as markers of TKI resistance, they did not play a greater role in activating NF-κB in TKI-resistant versus sensitive cells. Therefore, these proteins may alter the expression of additional peptide targets that have yet to be discovered, a subject for future investigation. Surprisingly, TKI-resistant cells demonstrated resistance to the proteasome inhibitor, bortezomib, despite having increased sensitivity to PSMD1 or PSMD3 knockdown in cell growth assays. However, we speculate that this may be due to the presence of STAT3. We have already reported activation of STAT3 at tyrosine 705 (pSTAT3^Y705^) in TKI resistance, and that dual targeting of STAT3 and BCR-ABL1 induced synthetic lethality in TKI-resistant CML [19, 20]. Importantly, the JAK1/STAT3 pathway was previously shown to alter proteasome inhibitor sensitivity in multiple myeloma [68], and therefore could possibly induce bortezomib resistance in the case of TKI-resistant CML. STAT3-mediated NF-κB activation likely also explains the reduced effects of PSMD1 or PSMD3 knockdown on NF-κB transcriptional activity that we observed in TKI-resistant versus TKI-sensitive cells, as seen in Fig. 7B, C.

The UPS is responsible for targeted protein degradation using three catalytic specificities: chymotrypsin-like, trypsin-like, and post-glutamyl hydrolytic [45]. It has long been established that the UPS plays an important role in NF-κB activation [69], and five members of the UPS were hits in a previously published shRNA library screen for TKI resistance [29]. Our data suggest that PSMD1 and PSMD3 are upregulated upon CML disease progression and regulate NF-κB protein expression, transcriptional activity, and cell growth. We speculate that the difference in sensitivity between CML and normal progenitors to knockdown of PSMD1 or PSMD3 may be due to differences in posttranslational modifications (PTMs), which could alter peptide targets in malignant versus normal tissues. It was previously reported that PSMD1 functions in the proteasome by acting as an ubiquitin receptor, binding of peptide substrates, and gate opening, which could be subject to several different PTMs (e.g., SUMOylation, N-acetylation, succinylation, and S-glutathionylation) [70]. As you can see in Fig. 3C, PSMD3 protein in CML CD34^+^ cells migrated farther on the gel than PSMD3 in cord blood, suggesting aberrant PTMs. In breast cancer, PSMD1 was shown to induce p53 protein degradation [30], whereas PSMD3 protected HER2 from degradation in HER2-positive breast cancers [31]. We propose that PSMD1 and PSMD3 protect NF-κB protein from degradation. In addition to PTMs that alter peptide substrates, another potential difference between CML and normal cells could be differential expression of proteins targeted for degradation. Saccani et al. [71] previously reported that the proteasome represses NF-κB transcriptional activity by direct polyubiquitination and degradation of p65 in the nucleus. These results indicate that proteasome-dependent degradation of NF-κB not only regulates its protein stability and abundance, but also actively promotes transcriptional termination. Future studies will explore PTMs and peptide targets of PSMD1 and PSMD3 in myeloid malignancies.

Similar to our findings, Fisher et al. reported increased phosphorylation and activation of NF-κB in myeloproliferative disorders, which was dependent on TNFα stimulation in hematopoietic stem and progenitor cells. Their results demonstrated that, in cells of patients with myelofibrosis and secondary AML, the NF-κB pathway was constitutively active and hypersensitive to cytokine stimulation compared with controls [72]. Tsvetkov et al. [73] reported that reduced mRNA expression of at least one 19S proteasome component, including *PSMD1*, *PSMD5, PSMD6*, or *PSMD10*, correlated with loss of TNFα/NF-κB signaling in myeloma cells that are resistant to the proteasome inhibitors, MG132, bortezomib, and carfilzomib. This agrees with our findings, in that shRNA-mediated knockdown of *PSMD1* or *PSMD3* in CML cells reduced NF-κB protein expression and transcriptional activity (Fig. 7), suggesting that PSMD1 and PSMD3 regulate NF-κB protein expression, rather than targeting the canonical signaling pathway. In contrast with our findings, low expression of proteasome components correlated with higher relapse rates and a lower overall survival in myeloma patients [73]. Since high levels of *PSMD3* mRNA correlated with a worse overall survival in AML [32], our data suggest tissue-specific roles for these proteins during malignant transformation of different tissue types. Additional studies will be required to determine if similar pathways are involved in the progression of other types of cancers. Altogether, our data suggest a role for PSMD1, PSMD3, and STAT3 in regulating NF-κB expression and activity in CML and TKI resistance, and implicate them as potential targets for the treatment of hematologic malignancies and possibly other forms of cancer.

## Methods

### Cell lines and patient samples

Details on the cell lines used in this study are available in Supplementary Methods. Primary mononuclear cells (MNCs) from discarded cord blood from the University Medical Center of El Paso (El Paso, TX, USA) were Ficoll-separated (Life Technologies, Carlsbad, CA, USA) and used for selection of the CD34^+^ population (StemCell Technologies Inc., Vancouver, Canada). CD34^+^ cells represent the disease-causing stem and progenitor population in CML. A purity of >90% was confirmed by flow cytometry using FITC anti-human CD34 (BioLegend Inc., San Diego, CA, USA). CD34^+^ cells from peripheral blood of CML patients or normal donors that were used for nucleocytoplasmic fractionation (see below) were obtained from the Centre for Haematology at Imperial College London (London, UK). Cells (2 × 10^6^) were lysed directly for use in molecular biology assays. This study was approved by the Texas Tech University Health Sciences Center El Paso Institutional Review Board (IRB) and by the Imperial College Research Ethics Committee. All patients gave informed consent and all experiments were performed in accordance with the Declaration of Helsinki.

### RNA sequencing (RNA-seq)

Detailed information on RNA-seq experiments is provided in Supplementary Methods. RNA-seq data generated in cell lines (GSE149623) were correlated with HG-U133 microarray data on CML CD34^+^ cells from TKI-resistant patients in a published data set (GSE14671) [27]. Genes were selected as having *p* < 0.01 and a fold change >1.5 in both studies. Heat maps were produced using the “gplots” package on R3.3.2 [74]. Gene-level expression data for *PSMD1* and *PSMD3* were also obtained from primary CML patient MNCs isolated from either peripheral blood or bone marrow and subjected to paired-end 2 × 150bp RNA-seq using the Illumina platform (part of a larger, separate study: Eide et al. manuscript in preparation). All patients exhibited BCR-ABL1-independent TKI resistance, and were separated by disease phase: CP-CML (*n* = 22), accelerated phase CML (AP-CML, *n* = 8), and BP-CML (*n* = 10). This study was approved by the Oregon Health & Science University IRB.

### Reverse transcription quantitative polymerase chain reaction (RT-qPCR)

Total RNA was extracted using the PureLink RNA Mini Kit by Invitrogen (Thermo Fisher Scientific, Waltham, MA, USA) and quantified using a NanoDrop^TM^ 2000 (Thermo Fisher Scientific). RT-qPCR was performed with the Luna Universal One-Step qPCR Kit (New England Biolabs, Ipswich, MA, USA) on a StepOnePlus Real-Time PCR System (Applied Biosystems, Foster City, CA, USA). *GUSB* mRNA levels were measured as a control; primer sequences are listed in Supplementary Table S1. Assays were performed in triplicate and relative expression was analyzed using the comparative cycle threshold method (2^−ΔΔCt^).

### Immunoblot

K562^S^ and K562^R^ cells and derivative lines were cultured as indicated, and resulting cells (10^6^) were lysed (4 °C; 30 min) in 1X RIPA buffer (Cell Signaling Technology Inc., Danvers, MA, USA) containing protease inhibitors (PMSF, Thermo Fisher Scientific) and phosphatase inhibitors (PhosSTOP, Roche, Basel, Switzerland). For primary samples, 3 × 10^4^ CD34^+^ cells were lysed directly in sample buffer. All samples were denatured (100 °C; 10 min) followed by SDS-PAGE and transfer to PVDF membranes. α/β-tubulin or β-actin was assessed as a loading control; antibodies are listed in Supplementary Table S2.

### Nucleocytoplasmic fractionation

For detection of total- and phospho-NF-κB p65 in CML cell lines and primary CD34^+^ cells, nuclear and cytoplasmic extracts were separated from 2 × 10^6^ cells prior to immunoblot using the Nuclear/Cytosol Fractionation Kit (BioVision, Inc., Milpitas, CA, USA). Lysis of membranes with preservation of nuclei was confirmed by light microscopy. Antibodies against α/β-tubulin and Lamin B1 were used to assess purity of the cytoplasmic and nuclear fractions, respectively (Supplementary Table S2). Densitometry was conducted using ImageJ (NIH, Bethesda, MD, USA).

### Constructs and lentivirus production

Lentiviral shRNA constructs targeting *PSMD1* (RefSeq: NM_002807.2), *PSMD3* (RefSeq: NM_002809.2), *STAT3* (RefSeq: NM_139276.2), and a non-targeting control vector (shNT) were purchased from Cellecta (Mountain View, CA, USA). The vector sequences were identified from our previously published shRNA library screen [29]. The pGreenFire NF-κB luciferase reporter system was purchased from System Biosciences, LLC (Palo Alto, CA, USA) (Supplementary Table S3). Detailed information on lentivirus production is provided in Supplementary Methods.

### Luciferase reporter assays

To detect endogenous NF-κB transcriptional activity, CML cell lines were lentivirally transduced with the pGreenFire1-NF-κB Lentivector reporter system (Supplementary Table S3) using the protocol outlined in Supplementary Methods. Stably transduced cells were selected by 10 days of culture in the presence of Geneticin™ (Life Technologies). CML cells containing the pGreenFire1-NF-κB reporter system were seeded at 5000 cells per well on a sterile black flat-bottom 96-well plate (Greiner Bio-One, Kremsmünster, Austria). Luciferase reporter activity was recorded 72 h following treatment using the ONE-Glo Luciferase Assay System (Promega Corporation, Madison, WI, USA) on a CLARIOstar^®^ Plus (BMG Labtech, Ortenberg, Germany).

### Apoptosis assays

Following doxycycline (100 ng/ml, 72 h) treatment with and without the indicated inhibitors, apoptosis was measured by staining cells with APC-conjugated AnnexinV (BioLegend Inc.) in combination with 7-aminoactinomycin D (eBioscience at Thermo Fisher Scientific). Resulting cells were analyzed using a BD FACSCanto II (BD Biosciences, San Jose, CA, USA), and the data were processed through FlowJo (Ashland, OR, USA).

### Time course assay

K562^S^ and K562^R^ cells expressing shPSMD1 or shPSMD3 were plated at a density of 1.0 × 10^5^ cells/ml ± doxycycline (100 ng/ml) to induce the knockdown. Cells were counted every other day for 9 days by trypan blue exclusion, with media changes and expansion as needed to maintain exponential growth.

### Colony formation assays

Methylcellulose colony formation assays were performed by plating of CML cell lines or primary CD34^+^ cells (10^3^ cells/dish) in 0.9% MethoCult (StemCell Technologies) as previously described [19]. Briefly, CML cell lines were plated in cytokine-free conditions ± imatinib (1.0 μM) and/or doxycycline (100 ng/ml) to induce shRNA-mediated knockdown. For GFP^+^CD34^+^ CML or cord blood cells expressing shPSMD1 or shPSMD3, cells were plated in the presence of 1X StemSpan^TM^ CD34^+^ Expansion Supplement (StemCell Technologies) ± doxycycline (100 ng/ml). Colonies were scored after 7–14 days of culture in a humidified chamber at 37 °C with 5% CO_2_.

### Enzyme-linked immunosorbent assay (ELISA)

To detect autonomous production of tumor necrosis factor alpha (TNFα) or IL-6, K562^S^ and K562^R^ cells were cultured at 1 × 10^6^ cells/ml for 24 h, and resulting supernatants were harvested and stored at −80 °C. TNFα was detected using the Human TNF-alpha Quantikine ELISA Kit (R&D Systems, Inc., Minneapolis, MN, USA, #DTA00D), and IL-6 was detected using the Human IL-6 Quantikine ELISA Kit (R&D Systems, Inc., #D6050) according to the manufacturer’s instructions. Signals were quantified on a CLARIOstar^®^ Plus (BMG Labtech) plate reader.

### Subcutaneous injections

Wild-type K562 cells were lentivirally transduced with shNT or shPSMD3 (Cellecta) and selected in puromycin (2 μg/ml, 72 h). To generate subcutaneous tumors, 3 × 10^6^ cells were mixed with 100 μl of Matrigel basement membrane matrix (Corning Inc., Corning, NY, USA #356234), and injected subcutaneously into the rear left and right flanks of 6–8-week-old nude mice (Jackson Laboratories #002019). Tumor size was measured every 3–4 days, and volume was calculated as previously described [75, 76]. Ten days after implantation, all mice were placed on 625 mg/kg doxycycline hyclate (Envigo Teklad, Indianapolis, IN, USA #TD.01306), and tumor size was measured over time. After 21 days, mice were euthanized and tumors removed for gross examination. Mice were not randomized, and the investigator was not blinded during image analysis. All experiments were performed with approval by the Institutional Animal Care and Use Committee at Texas Tech University Health Sciences Center El Paso.

### Statistical analyses

For all assays, three independent experiments were performed unless otherwise noted. A two-tailed Student’s *t* test was used for cell line, mouse, and cord blood data demonstrating equivocal variance. Patient sample RNA-seq data were analyzed by one-way analysis of variance in GraphPad Prism version 7.05. All error bars represent standard error of the mean, and a *p* value of <0.05 was considered statistically significant. For gene expression analyses, a *p* value of <0.01 was considered statistically significant. No statistical method was used for predetermination of sample size.

## Supplementary information

Supplementary Material
