# Supplementary material for: Proteasome 26S subunit, non-ATPases 1 (PSMD1) and 3 (PSMD3), play an oncogenic role in chronic myeloid leukemia by stabilizing nuclear factor-kappa B
Source: Oncogene. 2021 Mar 12;40(15):2697–710. doi: 10.1038/s41388-021-01732-6 (PMC7952820; doi:10.1038/s41388-021-01732-6)
Supplement: Supplementary file 1 — Supplementary Material [file 41388_2021_1732_MOESM1_ESM.pdf]

# Supplemental Information

## Proteasome 26S subunit, non-ATPases 1 (PSMD1) and 3 (PSMD3), play an oncogenic role in chronic myeloid leukemia by stabilizing nuclear factor-kappa B

Alfonso E. Bencomo-Alvarez[1], Andres J. Rubio[1], Idaly M. Olivas[2], Mayra A. Gonzalez[1], Rebecca Ellwood[3], Carme Ripoll Fiol[3], Christopher A. Eide[4], Joshua J. Lara[1], Christian Barreto-Vargas[5], Luis F. Jave-Suarez[6], Georgios Nteliopoulos[3,7], Alistair G. Reid[8], Dragana Milojkovic[3], Brian J. Druker[4], Jane Apperley[3], Jamshid S. Khorashad[3], Anna M. Eiring[1,2*]

[1]Center of Emphasis in Cancer, Department of Molecular and Translational Medicine, Paul L. Foster School of Medicine, Texas Tech University Health Sciences Center El Paso, El Paso, TX, USA; [2]Graduate School of Biomedical Sciences, Texas Tech University Health Sciences Center El Paso, El Paso, TX, USA; [3]Centre for Haematology, Department of Medicine, Imperial College London, London, UK; [4]Knight Cancer Institute, Division of Hematology/Medical Oncology, Oregon Health & Science University, Portland, OR, USA; [5]Universidad de Guadalajara, Guadalajara, Jalisco, México; [6]Instituto Mexicano del Seguro Social, Centro de Investigación Biomédica de Occidente, Guadalajara, Jalisco, México; [7]Department of Surgery and Cancer, Imperial College London, London, UK; [8]Molecular Pathology Unit, Liverpool Clinical Laboratories, Royal Liverpool University Hospital, Liverpool, UK.

[*]**Corresponding Author:** Anna M. Eiring, PhD; 5001 El Paso Drive, MSC 32002, Room 2112, El Paso, TX 79905; Ph: (915) 215-4812; E-Mail: anna.eiring@ttuhsc.edu

**Running Title:** PSMD1 and PSMD3 in chronic myeloid leukemia

**Keywords:** Chronic myeloid leukemia (CML); Tyrosine kinase inhibitor (TKI); Proteasome 26S subunit, non-ATPase 1 (PSMD1); Proteasome 26S subunit, non-ATPase 3 (PSMD3); Drug resistance.

## Supplemental Methods

**Cell lines and patient samples.** Parental K562 cells were purchased from American Type Culture Collection (Manassas, VA, USA) and cultured in RPMI medium (Life Technologies, Carlsbad, CA, USA) supplemented with 10% fetal bovine serum (FBS, Life Technologies Corporation), 2.0 mM L-glutamine (Life Technologies), and 100 U/ml penicillin/streptomycin (Life Technologies). To produce TKI-resistant K562 cells ($K562^R$), parental TKI-sensitive K562 cells ($K562^S$) were cultured in escalating concentrations of imatinib (0.1-1 μM, Selleck Chemicals, Houston, TX, USA) over the course of three months, as previously described (1, 2). Resulting $K562^R$ cells were confirmed to harbor exclusively native BCR-ABL1, and were cultured in the continuous presence of 1.0 μM imatinib unless otherwise noted. Where specified, cells were treated with the indicated concentrations of the proteasome inhibitor, bortezomib (Selleck Chemicals).

**RNA sequencing (RNA-seq).** *Cell lines:* RNA extraction was performed with the RNeasy mini kit (Qiagen, Hilden, Germany, #74104) with additional on-column DNase digestion, performed in triplicate per cell line. Samples were quantified using the Qubit 3.0 Fluorometer (Life Technologies), and RNA integrity (RIN) was validated with an Agilent 2100 Bioanalyzer using the Agilent RNA 6000 Nano kit (Agilent Technologies, Santa Clara, CA, USA). Samples with good quality (RINs > 9.90) were pooled prior to sequencing on the HiSeq 4000 sequencing platform using the HiSeq 3000/4000 SBS kit (Illumina, San Diego, CA, USA), generating ~64.5 million reads per sample for paired-end, 75 base pair sequencing with 150 cycles on the HiSeq 4000 (Illumina). Sequencing libraries were prepared using the TruSeq RNA Sample Preparation Kit (Illumina) according to manufactures instructions. These libraries were quantified with the Library Quantification Kit (KAPA) for Illumina systems and validated using a 4200 Tapestation Instrument (Agilent). The raw

sequencing data was checked for quality using FastQC version 0.11.5 (S. Andrews, Babraham Bioinformatics, Cambridge, UK). The data was then trimmed using Trimmomatic version 0.33 and the resulting quality filtered files were aligned to the human genome 38 cDNA (Ensembl, release 89) using Salmon 0.8.2 (3). Gene-level counts were obtained using 'TXimport' (version 1.8.0) (4) function in R. Gene level differential expression analysis between K562$^{S}$ and K562$^{R}$ samples was performed using the 'DESeq2' command in DESeq2 (version 1.20.0) (5). To assess the overall similarity between samples, the Euclidean distance was calculated on VST-transformed data (6), which is plotted as a heatmap showing clear separation between the two cell lines. Analyzed read counts were further sorted based on a 1.5-fold change cut off with $p<.01$ to establish the cohort of differentially expressed genes. Gene set enrichment analysis (GSEA) (7, 8) was performed using the differentially expressed genes identified by RNA-seq. RNA-seq data generated in cell lines were correlated with TKI-resistant patients from a published data set (9). Data sets were merged together based on gene names using the merge function on R3.3.2. Heat maps were produced using the 'gplots' package on R3.3.2 (10). Genes were selected as having a $p<0.01$ and a fold change >1.5 in both studies.

**Lentivirus production.** Lentivirus-producing 293FT cells (Life Technologies) were maintained in culture in DMEM plus 10% FBS, 2.0 mM L-glutamine, 100 U/ml penicillin/streptomycin, 1.0 mM sodium pyruvate (Life Technologies), and 0.1 mM MEM non-essential amino acids (Life Technologies). Constructs were packaged using psPAX2 and VSV.G (Supplemental Table S3). Viral particles were concentrated after binding to Polyethylene Glycol 8000 (1800 rpm, 45 min., 4ºC, Fisher Scientific, Hampton, NH, USA) and delivered to target cells by spinoculation (1). Cells were selected by fluorescence-activated cell sorting (FACS) or by treatment with 2 µg/ml puromycin

dihydrochloride (Thermo Fisher Scientific) or Geneticin™ Selective Antibiotic (G418 Sulfate) (Life Technologies). For doxycycline-inducible constructs, cells were treated ± doxycycline hyclate (100 ng/ml, 72 h, Gold Biotechnology Inc., St. Louis, MO, USA) to induce the knockdown.

## Supplemental Tables

| *Name* | *Forward Primer (5'>3')* | *Reverse Primer (5'>3')* |
|---|---|---|
| *GUSB* | GAAAATATGTGGTTGGAGAGCTCATT | CCGAGTGAAGATCCCCTTTTTA |
| *IL6* | CACAGACAGCCACTCACCTC | TTTTCTGCCAGTGCCTCTTT |
| *NF-κB p65* | ATCCCATCTTTGACAATCGTGC | CTGGTCCCGTGAAATACACCTC |
| *PSMD1* | GATCCAGGCACAGAAGCAAT | AGGAGGACAACCTGCTGATG |
| *PSMD3* | ATCACGCCCGGGTCTATGAG | ATGCCGAAGCGTAGCTGTCC |
| *TNFA* | TCAGCCTCTTCTCCTTCCTG | TCAGCTTGAGGGTTTGCTAC |

**Table S1. Primer sequences for RT-qPCR.** GUSB, glucuronidase beta; IL6, interleukin-6; NF-κB, nuclear factor-kappa B; PSMD1, proteasome 26S subunit, non-ATPase 1; PSMD3, proteasome 26S subunit, non-ATPase 3; TNFA, tumor necrosis factor alpha.

| *Name* | *Vendor* | *Catalog #* |
|---|---|---|
| *Rabbit monoclonal anti-NF-κB p65* | Cell Signaling Technology | 8242S |
| *Mouse monoclonal anti-phospho-NF-κB p65 (Ser536)* | Cell Signaling Technology | 3036S |
| *Mouse monoclonal anti-STAT3* | Cell Signaling Technology | 9139S |
| *Rabbit monoclonal anti-phospho-STAT3 (Tyr705)* | Cell Signaling Technology | 9145S |
| *Rabbit polyclonal anti-α/β-tubulin* | Cell Signaling Technology | 2148S |
| *Anti-rabbit IgG, HRP-linked Antibody* | Cell Signaling Technology | 7074 |
| *Anti-mouse IgG, HRP-linked Antibody* | Cell Signaling Technology | 7076 |
| *Rabbit polyclonal anti-PSMD1* | Life Technologies Inc. | PA1-973 |
| *Rabbit polyclonal anti-PSMD3* | Bethyl Laboratories Inc. | A303-826A |
| *Rabbit polyclonal anti-Ub (FL-76)* | Santa Cruz Biotechnology | sc-9133 |
| *Rabbit polyclonal anti-Lamin B1* | Abcam | ab16048 |
| *Mouse monoclonal anti-β-actin* | Sigma-Aldrich | A5441 |

**Table S2. Antibodies for immunoblot.** HRP, horseradish peroxidase; IgG, immunoglobulin G; NF-κB, nuclear factor-kappa B p65; PSMD1, proteasome 26S subunit, non-ATPase 1; PSMD3, proteasome 26S subunit, non-ATPase 3; STAT3, signal transducer and activator of transcription 3; Ub, ubiquitin.

| *Vector* | *Vendor* | *Catalog #* |
|---|---|---|
| *psPAX2* | Addgene | 12260 |
| *VSV.G* | Addgene | 14888 |
| *shPSMD1* | Cellecta, Inc. | CVSHC-PX |
| *shPSMD3* | Cellecta, Inc. | CVSHC-PX |
| *shSTAT3* | Cellecta, Inc. | CVSHC-PX |
| *Non-Targeting shRNA (shNT)* | Cellecta, Inc. | CVSHC-PX |
| *pGreenFire1-NF-κB Lentivector* | System Biosciences, LLC. | TR012PA-N |

**Table S3. Lentiviral vectors for virus production.** NF-κB, nuclear factor-kappa B (p65); psPAX2, empty backbone 2nd generation lentiviral packaging plasmid; shRNA, small hairpin ribonucleic acid; shPSMD1, shRNA vector targeting proteasome 26S subunit, non-ATPase 1; shPSMD3, shRNA vector targeting proteasome 26S subunit, non-ATPase 3; shSTAT3, shRNA vector targeting signal transducer and activator of transcription 3; VSV.G, envelope vesicular stomatitis virus G glycoprotein plasmid.

**Table S4. Top 30 differentially expressed genes ranked by fold-change in K562$^S$ versus K562$^R$ cells**

| Gene | Fold change | *P* | Function |
|---|---|---|---|
| **Upregulated Genes** | | | |
| *PTPN7* | 2463.26 | $2.15E^{-292}$ | Acts preferentially on tyrosine-phosphorylated MAPK1 |
| *COL6A3* | 2051.99 | $4.19E^{-143}$ | Encodes the alpha-3 of type VI collagen |
| *AMIGO2* | 1480.43 | $5.27E^{-146}$ | Adhesion molecule |
| *NTS* | 1449.38 | $1.30E^{-167}$ | Encodes a common precursor for two peptides, neuromedin N and neurotensin |
| *PRG2* | 1211.07 | $5.08E^{-245}$ | Comprises the crystalloid core of the eosinophil granule |
| *RYR2* | 1177.46 | $7.11E^{-137}$ | Encodes the ryanodine receptor |
| *ZC3H12C* | 1064.12 | $1.32E^{-173}$ | Role in TNFα signaling pathway via NF-κB |
| *PCED1B* | 917.27 | $4.17E^{-101}$ | Modification of biopolymers on the cell surface |
| *CCND1* | 818.80 | 0.00 | Regulator of CDK4 and CDK6 |
| *SYT10* | 778.30 | $3.64E^{-108}$ | $Ca^{2+}$ sensor |
| *TFAP2B* | 690.71 | $4.68E^{-111}$ | Transcriptional activator and repressor |
| *PTPRB* | 663.17 | $2.02E^{-136}$ | Signaling molecule that regulates a variety of cellular processes |
| *CELF2* | 599.54 | 0.00 | Regulates pre-mRNA alternative splicing |
| *DES* | 551.91 | $1.31E^{-171}$ | Encodes a muscle-specific class III intermediate filament |
| *COL15A1* | 510.94 | 0.00 | Encodes the alpha chain of type XV collagen |
| *CTAG2* | -4290.81 | $2.79E^{-255}$ | Encodes an autoimmunogenic tumor antigen |
| **Downregulated Genes** | | | |
| *PROX1* | -3634.08 | 0.00 | Member of the homeobox transcription factor family |
| *MAGEA1* | -2698.06 | 0.00 | Regulate the ubiquitination of proteins |
| *SMO* | -1137.20 | $2.65E^{-132}$ | A receptor for hedgehog proteins |
| *CAMK2D* | -970.64 | $6.35E^{-103}$ | Involved in calcium signaling |
| *CRMP1* | -853.20 | $5.39E^{-141}$ | Encodes a member of a family of cytosolic phosphoproteins |
| *ACSS1* | -791.14 | $5.07E^{-219}$ | Encodes a mitochondrial acetyl-CoA synthetase enzyme |
| *ARL4C* | -737.02 | $1.05E^{-139}$ | Regulates diverse cellular functions, such as vesicle traffic, endocytosis, and phospholipase D activity |
| *FCGRT* | -735.30 | $5.91E^{-114}$ | Encodes a receptor that binds the Fc region of monomeric immunoglobulin G |

| | | | |
|---|---|---|---|
| *RBMS1* | -617.60 | $5.32E^{-139}$ | Encodes a member of a small family of proteins which bind single stranded DNA/RNA |
| *ZNF711* | -614.24 | $1.39E^{-139}$ | Unknown |
| *SNCA* | -527.80 | $1.67E^{-190}$ | Member of the synuclein family |
| *DAB2IP* | -517.54 | $1.56E^{-103}$ | Tumor suppressor |
| *MSI1* | -509.24 | $1.92E^{-181}$ | Post-transcriptional gene regulation |
| *ZNF551* | -474.00 | $3.04E^{-205}$ | Role in gene expression and nucleic acid binding |

**Table S5. Genes commonly dysregulated in TKI-resistant cell lines and patient samples**

| Gene | Fold change $CD34^+$ cells | Fold change cell lines | *P* $CD34^+$ cells | *P* cell lines | Function |
|---|---|---|---|---|---|
| **Upregulated Genes** | | | | | |
| *CFH* | 1.53 | 4.16 | 0.001 | 0.00 | Member of the Regulator of Complement Activation (RCA) gene cluster |
| *MARCKS* | 2.04 | 94.52 | 0.002 | 0.00 | Role in cell shape, motility, and secretion |
| *C10orf128* | 2.49 | 70.94 | 0.001 | 0.00 | Protein coding gene |
| *ZC3H12C* | 1.89 | 1064.12 | 0.005 | $1.32E^{-173}$ | Role in TNFα signaling pathway via NF-κB |
| *NEK7* | 1.65 | 2.77 | 0.01 | $3.54E^{-82}$ | Role in mitosis |
| *FAM3C* | 1.56 | 2.59 | 0.003 | $7.05E^{-63}$ | Encodes a secreted protein with a GG domain |
| *RAB27B* | 2.20 | 433.85 | 0.001 | $5.61E^{-70}$ | Involved in vesicular fusion and trafficking |
| *MAP2K4* | 1.55 | 2.59 | 0.008 | $5.62E^{-54}$ | Encodes a member of the mitogen-activated protein kinase (MAPK) family |
| *TAOK1* | 1.71 | 4.08 | 0.005 | $3.40E^{-219}$ | Ste20-related mitogen-activated protein kinase kinase kinases (MAP3Ks) |
| *ITPR1* | 1.57 | 4.93 | 0.00 | $1.53E^{-88}$ | Encodes an intracellular IP3-gated calcium channel |
| *GPATCH2* | 1.82 | 19.09 | 0.002 | $5.53E^{-54}$ | Involved in RNA processing |
| *TMEM163* | 2.10 | 23.55 | 0.01 | $8.62E^{-30}$ | A zinc transporter |
| *TMEM45A* | 2.22 | 9.46 | 0.007 | $6.80E^{-11}$ | Transmembrane protein |
| *PSMD1* | 2.36 | 4.56 | 1.17E-32 | $1.19E^{-31}$ | Non-ATPase subunit of the 19S proteasome |
| *CYB5B* | 1.97 | 1.61 | 0.004 | $1.87E^{-31}$ | Cytochrome |
| *PTGS2* | 2.53 | 86.01 | 0.036 | $2.82E^{-28}$ | Involved in prostaglandin biosynthesis |
| *TANC1* | 1.96 | 7.90 | 0.005 | $1.09E^{-37}$ | Scaffold component |
| *PSMD3* | 2.26 | 5.40 | 3.91E-27 | $3.37E^{-26}$ | Non-ATPase subunit of the 19S proteasome |
| *DDX54* | 1.7 | 5.77 | 3.99E-23 | $2.98E^{-22}$ | DEAD box RNA helicase |
| *BCL11A* | 1.98 | 4.36 | 0.01 | $3.85E^{-06}$ | Encodes a zinc binding protein |
| *MED31* | 1.81 | 1.79 | 0.00 | $2.16E^{-05}$ | Mediator of RNA polymerase II transcription subunit 31 |
| *GNAI1* | 1.53 | 14.62 | 0.006 | $1.62E^{-14}$ | Hydrolyses GTP |
| *SCN9A* | 2.32 | 1.97 | 0.00 | $6.47E^{-20}$ | Encodes a voltage-gated sodium channel |
| *NHLRC3* | 1.56 | 1.67 | 0.005 | $6.00E^{-06}$ | Protein modification through ubiquitination |

| | | | | | |
|---|---|---|---|---|---|
| *RBM25* | 1.65 | 1.55 | 0.008 | $8.39E^{-14}$ | A regulator of alternative pre-mRNA splicing |
| *TRPS1* | 2.28 | 4.16 | 0.011 | $5.62E^{-12}$ | Encodes a transcription factor that represses GATA-regulated genes |
| *MLLT3* | 1.51 | 5.53 | 0.004 | $9.47E^{-05}$ | Potent leukemia oncogene |
| *BAZ2B* | 1.66 | 1.50 | 0.005 | $2.98E^{-06}$ | Involved in chromatin remodeling complexes |
| *MEIS1* | 1.87 | 1.71 | 0.003 | 0.0019797 | Involved in transcriptional regulation |
| *HIST1H3D* | 2.51 | 3.00 | 0.006 | $2.47E^{-14}$ | Involved in gene-silencing and heterochromatin formation |
| **Downregulated Genes** | | | | | |
| *SLC26A11* | -1.57 | -2.37 | 0.003 | 0.000943404 | Maintains homeostasis and intracellular electrolyte balance |
| *ARL4C* | -2.37 | -737.02 | 0.072 | $1.05E^{-139}$ | Regulates diverse cellular functions, such as vesicle traffic, endocytosis, and phospholipase D activity |
| *MS4A3* | -2.01 | -6.16 | 0.01 | $3.90E^{-38}$ | Plays a role in signal transduction |
| *S100A10* | -1.88 | -3.67 | 0.004 | 0.000434535 | Regulate cell cycle |
| *HOMER3* | -1.59 | -4.45 | 0.002 | $4.28E^{-08}$ | Scaffold protein |
| *SPTLC2* | -1.55 | -1.52 | 0.003 | $4.02E^{-12}$ | Encodes a long chain base subunit of serine palmitoyltransferase |
| *SLC22A4* | -1.85 | -13.99 | 0.004 | $7.79E^{-154}$ | Polyspecific organic cation transporter |

## Supplemental Figures

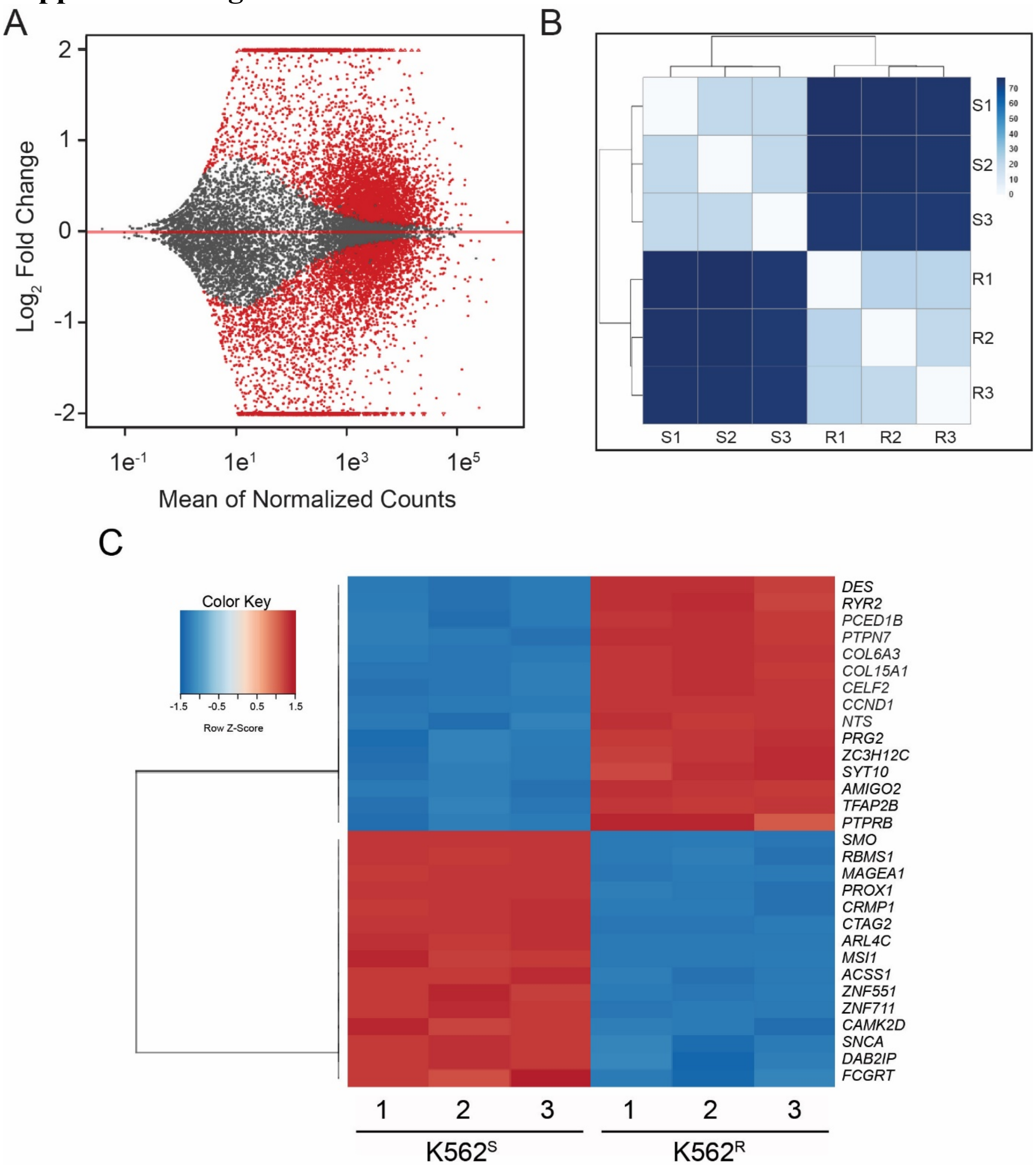


**Figure S1. MA (ratio intensity) and heatmap plots for RNA-seq data comparing TKI-resistant K562$^R$ cells to TKI-sensitive K562$^S$ controls. A.** MA plot shows the overall distribution of differentially expressed genes. Log fold-change represents the 'M' which stands for "minus," where subtraction of log values is equivalent to the log of the ratio. The mean of normalized counts represents the 'A' for "average." **B.** To assess the overall similarity between samples, the Euclidean distance was calculated on VST-transformed data, and plotted as a heat map showing clear separation between the two cell lines (S, sensitive; R, resistant). **C.** The heat maps show the top 30 differentially expressed genes in K562$^R$ versus K562$^S$ cells.

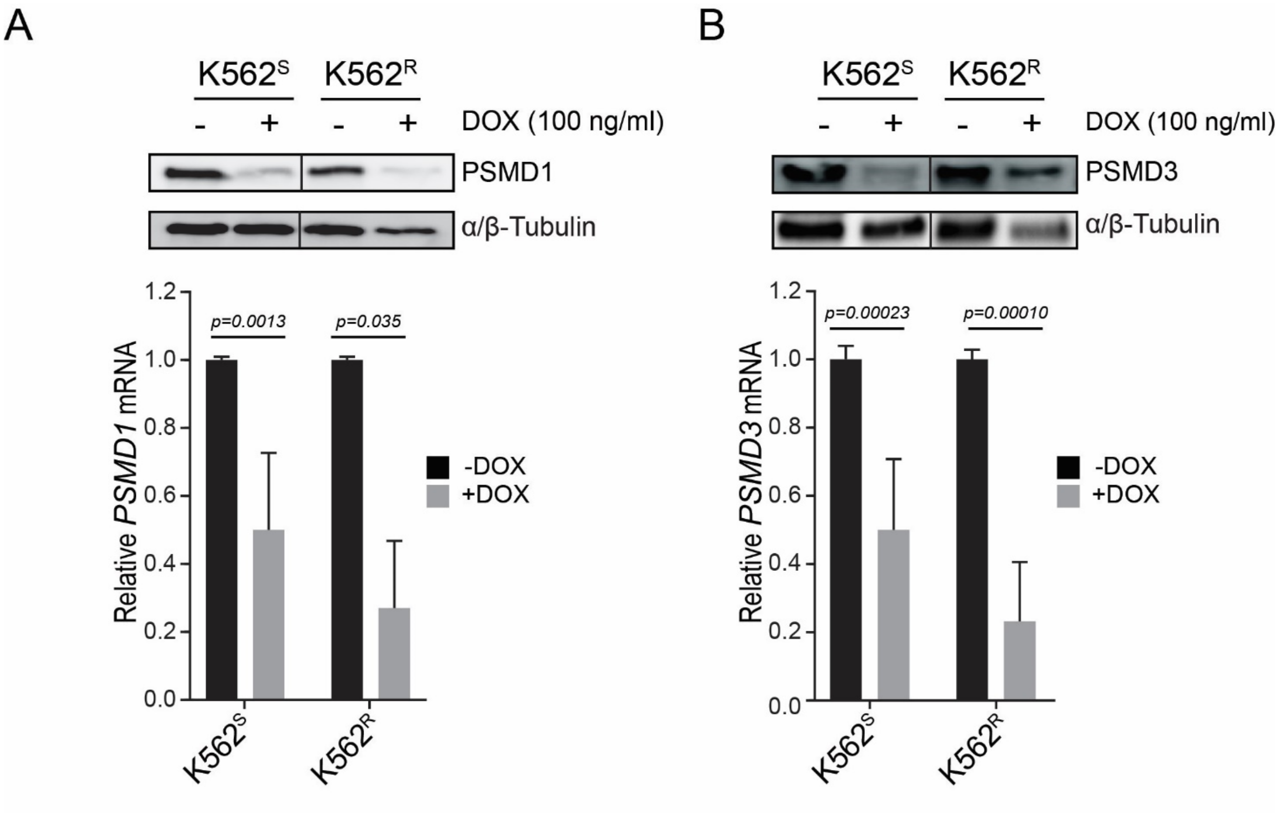


**Figure S2. Confirmation of shRNA-mediated knockdown of PSMD1 (shPSMD1) and PSMD3 (shPSMD3). A-B.** shRNA-mediated knockdown of PSMD1 (n=5) (**A**) or PSMD3 (n=4) (**B**) was confirmed at the mRNA level by RT-qPCR (*bottom*) and the protein level by immunoblot analyses (*top*). Error bars represent standard error of the mean (SEM). DOX, doxycycline; PSMD1, proteasome 26S subunit non-ATPase 1; PSMD3, proteasome 26S subunit non-ATPase 3.

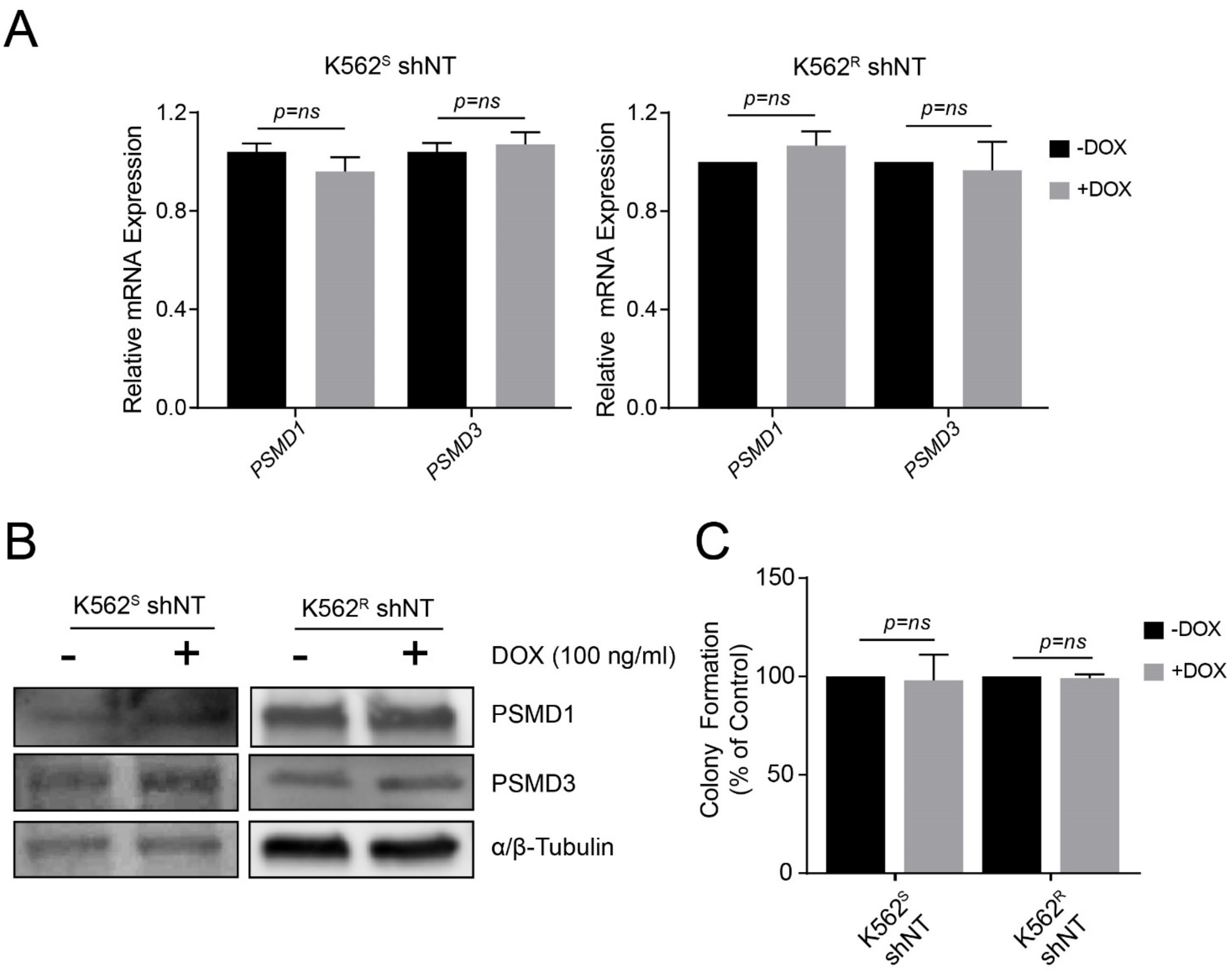


**Figure S3. The non-targeting shRNA (shNT) control vectors has no effect on parental K562 cells. A.** Bar graphs show the effects of shNT on *PSMD1* and *PSMD3* mRNA levels in parental $K562^S$ cells (*left*) and TKI-resistant $K562^R$ cells (*right*) in the presence and absence of doxycycline (n=3). **B.** Immunoblot confirms that shNT has no effect on PSMD1 or PSMD3 protein levels in $K562^S$ (*left*) or $K562^R$ cells (*right*). **C.** Bar graphs show the effects of shNT on colony forming ability in $K562^S$ or $K562^R$ cells. DOX, doxycycline; PSMD1, proteasome 26S subunit non-ATPase 1; PSMD3, proteasome 26S subunit non-ATPase 3; shNT, small hairpin non-targeting control RNA.

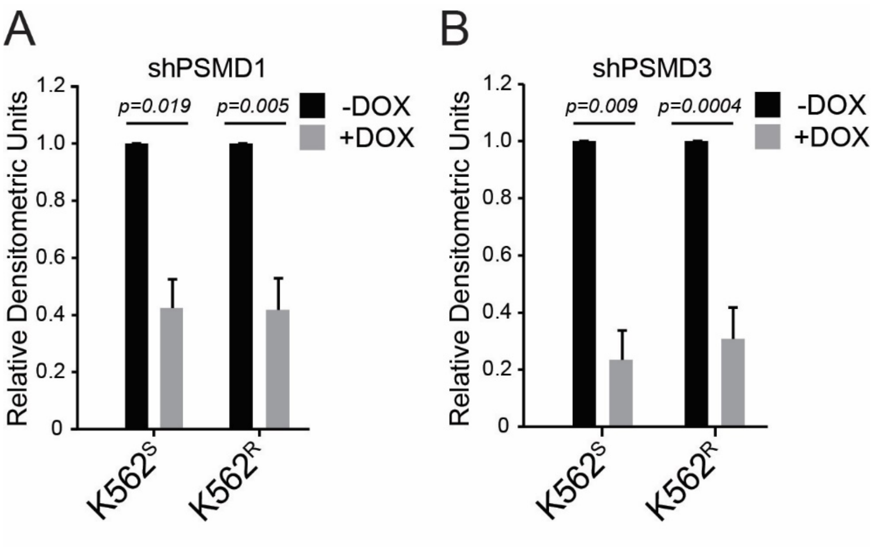


**Figure S4. Knockdown of PSMD1 or PSMD3 results in a similar level of reduced NF-κB protein expression comparing TKI-sensitive versus TKI-resistant K562 cells. A-B.** shRNA-mediated knockdown of PSMD1 (n=3, **A**) or PSMD3 (n=3, **B**) reduced relative NF-κB protein levels by a similar extent in $K562^S$ versus $K562^R$ cells in the presence of doxycycline (100 ng/ml, 72 h). Error bars represent SEM. DOX, doxycycline.

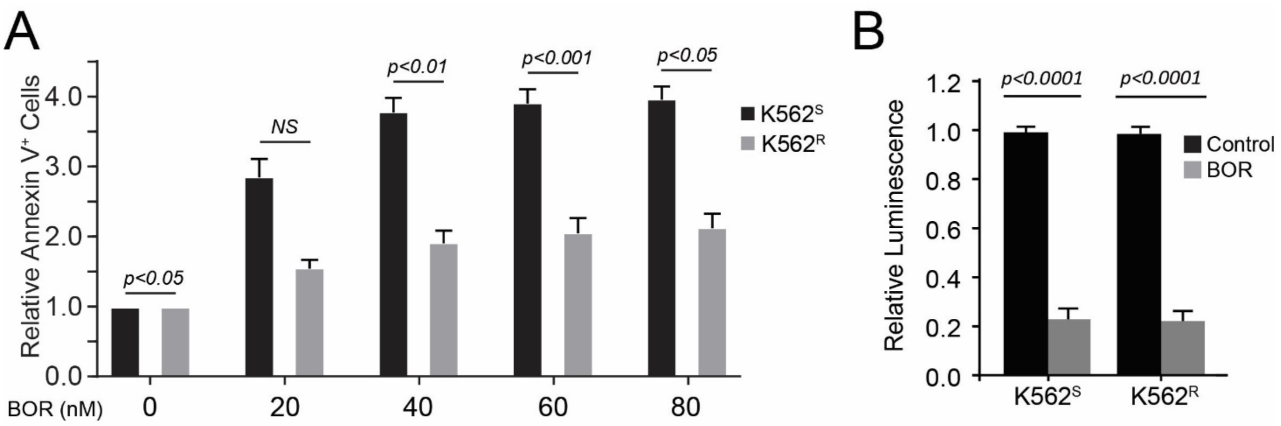


**Figure S5. TKI-resistant $K562^R$ cells demonstrate cross-resistance to the proteasome inhibitor, bortezomib. A.** Bar graph shows relative Annexin V-positive $K562^S$ and $K562^R$ cells (n=3 per group) upon treatment with graded doses of bortezomib (0-80 nM). **B.** Bar graph shows the effect of bortezomib treatment (0-80 nM) on NF-κB luciferase reporter activity in $K562^S$ and $K562^R$ cells (n=3 per group). BOR, bortezomib.

## Supplementary References


1. Eiring AM, Page BD, Kraft IL, Mason CC, Vellore NA, Resetca D, et al. Combined STAT3 and BCR-ABL1 inhibition induces synthetic lethality in therapy-resistant chronic myeloid leukemia. Leukemia. 2015;29(3):586-97.

2. Mahon FX, Deininger MW, Schultheis B, Chabrol J, Reiffers J, Goldman JM, et al. Selection and characterization of BCR-ABL positive cell lines with differential sensitivity to the tyrosine kinase inhibitor STI571: diverse mechanisms of resistance. Blood. 2000;96(3):1070-9.

3. Patro R, Duggal G, Love MI, Irizarry RA, Kingsford C. Salmon provides fast and bias-aware quantification of transcript expression. Nat Methods. 2017;14(4):417-9.

4. Soneson C, Love MI, Robinson MD. Differential analyses for RNA-seq: transcript-level estimates improve gene-level inferences. F1000Research. 2015;4:1521.

5. Love MI, Huber W, Anders S. Moderated estimation of fold change and dispersion for RNA-seq data with DESeq2. Genome Biol. 2014;15(12):550.

6. Zhao S, Guo Y, Sheng Q, Shyr Y. Advanced heat map and clustering analysis using heatmap3. BioMed Res Int. 2014;2014:986048.

7. Mootha VK, Lindgren CM, Eriksson KF, Subramanian A, Sihag S, Lehar J, et al. PGC-1alpha-responsive genes involved in oxidative phosphorylation are coordinately downregulated in human diabetes. Nat Genet. 2003;34(3):267-73.

8. Subramanian A, Tamayo P, Mootha VK, Mukherjee S, Ebert BL, Gillette MA, et al. Gene set enrichment analysis: a knowledge-based approach for interpreting genome-wide expression profiles. Proc Natl Acad Sci USA. 2005;102(43):15545-50.

9. McWeeney SK, Pemberton LC, Loriaux MM, Vartanian K, Willis SG, Yochum G, et al. A gene expression signature of CD34+ cells to predict major cytogenetic response in chronic-phase chronic myeloid leukemia patients treated with imatinib. Blood. 2010;115(2):315-25.

10. Warnes GR, Bolker B, Bonebakker L, Gentleman R, Huber W, Liaw A, et al. Package gplots. Available from https://CRAN.R-project.org/package=gplots. 2016.
